# Correlation Between Quantitative Hepatitis B Surface Antigen and Hepatitis B Virus Deoxyribonucleic Acid Levels in Hepatitis B e Antigen-Positive and Hepatitis B e Antigen-Negative Chronic Hepatitis B Patients

**DOI:** 10.5152/tjg.2020.19612

**Published:** 2023-04-01

**Authors:** Viva Finhar Insani Nirmala, Aryati Aryati, Hani Susianti, Syifa Mustika

**Affiliations:** 1 Department of Clinical Pathology, Airlangga University Faculty of Medicine, Dr. Soetomo General Hospital, Surabaya, Indonesia; 2 Department of Clinical Pathology, Brawijaya University Faculty of Medicine, Dr. Saiful Anwar General Hospital, Malang, Indonesia; 3 Division of Gastroentero-hepatology, Department of Internal Medicine, Brawijaya University Faculty of Medicine, Dr. Saiful Anwar General Hospital, Malang, Indonesia

**Keywords:** Chronic hepatitis B, cut-off value, HBV DNA, quantitative HBsAg

## Abstract

**Background:**

**:** This study aimed to analyze the relationship between quantitative hepatitis B surface antigen and hepatitis B virus deoxyribonucleic acid in hepatitis B e antigen-positive and hepatitis B e antigen-negative chronic hepatitis B patients and to determine the best cut-off value for quantitative hepatitis B surface antigen to predict high hepatitis B virus deoxyribonucleic acid levels (≥2000 IU/mL).

**Methods:**

Ninety-seven sera from chronic hepatitis B patients were collected in this study. Hepatitis B virus deoxyribonucleic acid levels were quantified by real-time polymerase chain reaction. Quantitative hepatitis B surface antigen and hepatitis B e antigen levels were determined by two-site sandwich chemiluminescence immunoassay. Alanine transaminase levels were measured by the International Federation of Clinical Chemistry-approved methods.

**Results:**

A significant correlation between quantitative hepatitis B surface antigen and hepatitis B virus deoxyribonucleic acid levels was observed in hepatitis B e antigen-positive group (*r* = 0.453, *P* = .002), but not in hepatitis B e antigen-negative group (*r* = 0.117, *P* = .454). No significant correlation between quantitative hepatitis B surface antigen and alanine transaminase was found in the hepatitis B e antigen-positive group (*r* = 0.521, *P* = .241). However, a significant correlation was shown between quantitative hepatitis B surface antigen and alanine transaminase levels in the hepatitis B e antigen-negative group (*r* = 0.455, *P* = .001). The best cut-off value of quantitative hepatitis B surface antigen for predicting high hepatitis B virus deoxyribonucleic acid levels was 3.422 × 10^3^ IU/mL.

**Conclusion:**

Correlation between quantitative hepatitis B surface antigen and hepatitis B virus deoxyribonucleic acid levels is significant in the hepatitis B e antigen-positive group. Quantitative hepatitis B surface antigen can be used to predict high hepatitis B virus deoxyribonucleic acid levels in the hepatitis B e antigen-positive group.

## Introduction

Hepatitis is a public health problem in developing countries worldwide, including Indonesia. Globally, an estimated 2 billion people have become infected with the hepatitis B virus (HBV) and around 240 million people suffered from chronic HBV infection.^1^ Indonesia is a country with a moderate to high prevalence of such infection, with 2.5%-10% of the population being chronic carriers of HBV.^2^ Patients with HBV infection are having a high risk of end-stage liver complications. Hence, it is very important to monitor the therapeutic response in chronic hepatitis B (CHB) cases.^3^ To date, measurement of HBV deoxyribonucleic acid (DNA) levels, which is expensive, time-consuming, and requires trained personnel, is a gold standard evaluation method. Quantitative hepatitis B surface antigen (qHBsAg) is currently being considered as an alternative biomarker because it is simple, inexpensive, rapid, and reflects the transcription activity of the covalently closed circular DNA (cccDNA) in nuclei of liver cells.^4-7^ Various studies related to the clinical use of qHBsAg and its relationship with HBV DNA have been carried out, but the results are still contradictory or controversial and studies on the correlation between qHBsAg and HBV DNA levels in Indonesia have been scarce.

Therefore, this study was conducted to find a correlation between qHBsAg and HBV DNA levels in hepatitis B e antigen (HBeAg)-positive and HBeAg-negative CHB patients and to determine the best cut-off value for qHBsAg in predicting high HBV DNA levels.

## Materials and Methods

### Subjects and Study Design

This cross-sectional study was conducted for 3 months, starting from February to April 2019. The measurement of HBV DNA and ALT levels was carried out at the Central Laboratory of Dr. Saiful Anwar General Hospital, Malang, and the determination of qualitative HBsAg, qHBsAg levels, and HBeAg was performed at the Indonesian Red Cross, Bangil Branch, Pasuruan. 

Sera from patients with a diagnosis of CHB whose complete medical records were available and did not meet the exclusion criteria were included in this study. The exclusion criteria included a co-infection with hepatitis C virus (HCV) or human immunodeficiency virus (HIV) and a diagnosis of hepatoma or liver cirrhosis. The diagnosis of CHB was based on HBsAg positivity for >6 months. 

During the study period, a stored blood sample from a patient who had undergone HBV DNA quantification was centrifuged and the serum obtained was tested for qualitative HBsAg. Sera from patients who met the selection criteria were divided into 3 aliquots and then kept at −70°C. These aliquots will be subjected to quantitative HBsAg, HBeAg, and alanine transaminase (ALT) determination when the minimum sample size had been achieved. Based on the results obtained, samples can be subsequently classified into HBeAg-positive and HBeAg-negative groups.

### Hepatitis B Surface Antigen Quantification

Hepatitis B surface antigen was quantified by two-site sandwich chemiluminescence immunoassay (CLIA) using a CL-series CLIA analyzer and HBsAg kit (catalog no: HBsAg131) from Mindray Bio-Medical Electronics Co Ltd, Shenzen, China, according to the manufacturer’s instructions. Results were expressed in IU/mL. This assay was calibrated against the WHO standard and allowed the quantification of HBsAg from 0.05 to 250 IU/mL. Samples with HBsAg levels >0.08 IU/mL were considered as positive. Samples with HBsAg levels >250 IU/mL required a 1 : 500 dilution with the diluent as recommended by the manufacturer.

### Hepatitis B e Antigen Measurement

Hepatitis B e antigen was measured by a two-site sandwich CLIA using a CL-series CLIA analyzer and HBeAg kit (catalog no: HBeAg131) from Mindray Bio-Medical Electronics Co Ltd, Shenzen, China, according to the manufacturer’s instructions. Samples with a cut-off index (COI) ≥ 1.00 were defined as positive and COI <1 as negative.

### Hepatitis B Virus Deoxyribonucleic Acid Quantification

Hepatitis B virus DNA was quantified by real-time polymerase chain reaction (PCR) using Exycycler™ 96 and AccuPower® HBV Quantitative PCR kit (catalog no: HBV DNA 1111) from Bioneer Corp., Daejeon, South Korea. High-purity viral DNA was extracted using ExiPrep™ 16 Dx and ExiPrep™ Dx Viral DNA/ribonucleic acid (RNA) (catalog no: K-4471), also from Bioneer Corp., Daejeon, South Korea. Both procedures were performed according to the manufacturer’s instructions. Results were expressed in IU/mL. Samples with HBV DNA levels ≥ 15 IU/mL were considered positive. Samples with HBV DNA levels ≥2000 IU/mL were considered high.^8^

### Statistical Analysis

Quantitative variables were expressed as mean ± standard deviation (SD) and qualitative variables were expressed as numbers with percentages. The Kolmogorov–Smirnov test was applied to assess the normality of qHBsAg and HBV DNA levels distribution. Non-parametric two-tailed Spearman’s test was conducted to determine the correlation between qHBsAg and HBV DNA levels. Receiver operating characteristic (ROC) curve analysis was performed to find the best cut-off value of qHBsAg in predicting HBV DNA levels. Microsoft Excel software was used to calculate numerical data. SPSS for Windows software (version 17.0; Chicago, IL, USA) was used to perform all statistics. *P* values less than .05 were regarded as statistically significant. 

## Results

A total number of 121 patients had requested HBV DNA quantification and their blood samples had been tested for qualitative HBsAg during the study period. Of these, 24 patients were excluded because 1 patient had a hepatoma, and 23 patients suffered from liver cirrhosis. Out of 97 patients included in this study, 47 (48.5%) were HBeAg positive while 50 (51.5%) were HBeAg negative. Both patient groups were dominated by males. The average age of the patients was 41.10 ± 13.111 years. There was a significant difference in qHBsAg, HBV DNA, and ALT levels between HBeAg-positive and HBeAg-negative patients (Table 1).

The correlation between qHBsAg and HBV DNA levels was significant in all patients and HBeAg-positive patients (*r* = 0.345, *P* = .001 and *r* = 0.453, *P* = .002, respectively), but insignificant in HBeAg-negative patients, whereas the correlation between qHBsAg and ALT levels was significant in all patients and HBeAg-negative patients (*r* = 0.311, *P* = .002 and *r* = 0.455, *P* = .001, respectively), but insignificant in HBeAg-positive patients. These correlations are shown in Table 2.

**Figure 1.**
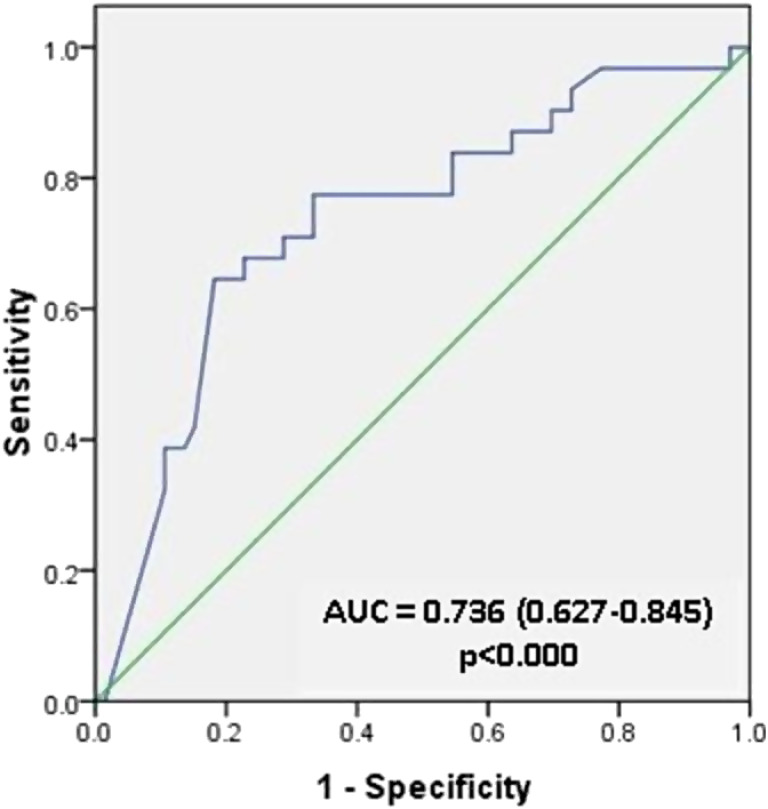
Receiver operating characteristic (ROC) curve for qHBsAg levels to predict high HBV DNA levels in CHB patients.

The ROC curve analysis revealed that the area under the curve (AUC) of qHBsAg to predict high HBV DNA levels was 0.736 (95% CI 0.627-0.845; *P *< .000), and the best cut-off value was 3.422 × 10^3^ IU/mL, with a sensitivity of 71% and a specificity of 71.2% (Figure 1).

## Discussion

In the present study, the mean age of CHB patients was 41.1 years old and the majority of them were males, which is in agreement with other studies.^9-11^ The average qHBsAg level was 3043.73 IU/mL. This result corresponds with the level of 4021 ± 2305 IU/mL reported by Ganji et al^12^ in Iran but in contrast to the level of 1.16 × 10^5^ IU/mL stated by Primadharsini and Wibawa^13^ in Bali. Disagreement in these qHBsAg levels may be caused by different phases of the disease at which the samples were collected. A study by Karra et al^14^ showed that qHBsAg level is 10^5^ IU/mL in the immune tolerant phase, 10^4^ IU/mL in the immune clearance phase, 10^3^ IU/mL in the inactive carrier phase, and 10^3^ IU/mL in the immune clearance phase.^14^ Based on Karra et al’s result, it is possible that sample collection in Primadharsini and Wibawa’s study was mostly carried out in the immune tolerant phase, whereas in our study, it was largely performed during the inactive carrier phase. The average HBV DNA levels of all patients in this study were 1.86 × 10^7^ IU/mL, which is similar to the levels of 5.9 × 10^7^ copies/mL and 7.53 × 10^6^ copies/mL observed by Primadharsini and Wibawa (2013) in HBeAg-positive and HBeAg-negative patients, respectively.^13^

There was a significant correlation between qHBsAg and HBV DNA levels in all CHB patients. However, a stronger correlation was noted in HBeAg-positive patients. These findings can be explained by an increase in cccDNA transcription activity. When HBV invades liver cells, the viral DNA will be converted into covalently closed circular DNA (cccDNA) and undergo transcription in the nuclei. Transcription products will be transferred to the cytoplasm to be translated into the nucleocapsid form and precore antigen C (C, pre-C), polymerase (P), HBsAg, transcriptional transactivating proteins (X), and pregenomic ribonucleic acid (pgRNA). An intact nucleocapsid with HBV DNA will be reconstructed and will be assembled together with HBsAg in the endoplasmic reticulum to form infectious complete viral particles.^15,16^ As cccDNA transcription activity increases, HBsAg, hepatitis B core antigen (HBcAg), and HBeAg secretion and HBV DNA replication will increase, leading to the development of a positive correlation between HBsAg and HBV DNA levels in HBeAg-positive patients. Several prior studies also reported a significant correlation between qHBsAg and HBV DNA levels.^4,8,10,13,16^

No significant correlation was observed between qHBsAg and HBV DNA levels in the HBeAg-negative patients. According to the European Association for the Study of the Liver (EASL), HBeAg-negative patients can be divided into 2 phases: inactive carrier and reactivation or HBeAg-negative CHB. The inactive carrier phase is indicated by the absence of HBeAg, low levels of HBsAg (usually <1000 IU/mL), presence of anti-HBe, undetectable or low levels of HBV DNA (<2000 IU/mL, in some cases, it can be between 2000 and 20 000 IU/mL), normal ALT levels, and absence of necroinflammation in liver tissue. Hepatitis B e antigen-negative CHB phase is characterized by the absence of HBeAg, fluctuating or persistently increased ALT levels, moderate HBsAg levels, moderate to high HBV DNA levels (>2000 IU/mL, often lower than in the HBeAg-positive group) which can persist or fluctuate, and moderate to severe liver damage.^17^ In the present study, the HBeAg-negative group had high HBV DNA levels and moderate qHBsAg levels. There are 2 possibilities that might cause these outcomes. One is resistance to antiviral drugs. The second possibility is that this group actually consists of HBeAg-positive patients (immune tolerant phase) whose HBeAg was negative due to a mutation in the precore region of the viral genome that inhibits the formation of HBeAg.

A significant correlation was also found between qHBsAg and ALT levels in all CHB patients with a stronger correlation seen in HBeAg-negative patients. These results were in accordance with the criteria established by the EASL for HBeAg-negative patients. When the body’s non-specific and specific immune responses have been able to eliminate the virus from the liver cells and blood circulation, the viral load will decrease which is then followed by a reduction in transcription activity of cccDNA, resulting in diminished secretion of HBsAg, HBeAg, and HBcAg.

We performed an ROC curve analysis to determine the best cut-off value for HBsAg in predicting HBV DNA levels using an HBV DNA level of ≥2000 IU/mL as the standard value. This value was chosen because the EASL guideline recommends an HBV DNA threshold of ≥2000 IU/mL to begin treatment of CHB regardless of the HBeAg status.^18^ Virological response is defined as undetectable HBV DNA as measured by sensitive PCR assay, which is an HBV DNA level of <2000 IU/mL.^17^ The AUC was 73.6% and the best cut-off value was 3422 IU/mL. These results are similar to the study conducted by Gupta^8^ which stated that the qHBsAg cut-off value to predict HBV DNA level of ≥2000 IU/mL is 3360 IU/mL.^8^

Our study has several limitations. The cross-sectional design of this study limited the chance to conduct serial measurements of HBsAg and HBV DNA levels in various phases of the disease. Much data related to diseases that can cause comorbidities in the liver were not available in the patients’ medical records, which may affect the homogeneity of the study population. Moreover, blood samples used in this study were not obtained from naïve CHB patients but from patients who were undergoing treatment, so the patients’ clinical presentations did not fit the disease phase in naïve cases.

In conclusion, quantitative HBsAg correlated significantly with HBV DNA and can be utilized to predict its levels in HBeAg-positive CHB patients. A level of qHBsAg ≥3422 IU/mL may be used to distinguish high HBV DNA (≥2000 IU/mL) from low HBV DNA (<2000 IU/mL). 

## Figures and Tables

**Table 1.** Demographic and Laboratory Characteristics of the Patients

**Variables**	**All Patients (N = 97)**	**HBeAg-Positive Patients (N = 47)**	**HBeAg-Negative Patients (N = 50)**	***P***
Sex (M/F)	60(61.9)/37(38.1)	24(51.1)/23(48.9)	36(72.0)/14(28.0)	.034*
Age (years)	41.10 ± 13.111	34.96 ± 11.438	46.88 ± 11.987	<.001*
qHBsAg (IU/mL)	3043.74 ± 2769.809	3783.84 ± 2718.998	2348.06 ± 2659.133	.016*
HBV DNA (IU/mL)	1.86 × 10^7^ ± 5.79 × 10^7^	2.96 × 10^7^ ± 6.96 × 10^7^	7.08 × 10^7^ ± 4.01 × 10^7^	.048*
ALT (U/L)	34.23 ± 35.128	40.28 ± 41.182	28.54 ± 27.520	.029*

*Significant difference at *P *< .05. Data are presented as N (%) or mean ± standard deviation. qHBsAg, quantitative hepatitis B surface antigen; HBV DNA, hepatitis B virus deoxyribonucleic acid; ALT, alanine transaminase.

**Table 2.** Correlations Between qHBsAg, HBV DNA, and ALT Levels

**Variables**		**qHBsAg**	
		***r***	***P***
HBeAg-positive patients	HBV DNA	0.453	.002*
	ALT	0.096	.521
HBeAg-negative patients	HBV DNA	0.117	.454
	ALT	0.455	.001*
All patients	HBV DNA	0.345	.001*
	ALT	0.311	.002*

*Significant correlation at *P *< .05. qHBsAg, quantitative hepatitis B surface antigen; HBV DNA, hepatitis B virus deoxyribonucleic acid; ALT, alanine transaminase.
